# Genetic analysis of influenza B viruses isolated in Uganda during the 2009–2010 seasons

**DOI:** 10.1186/1743-422X-10-11

**Published:** 2013-01-05

**Authors:** Denis K Byarugaba, Bernard Erima, Monica Millard, Hannah Kibuuka, Lukwago L, Josephine Bwogi, Derrick Mimbe, Edison A Mworozi, Bridget Sharp, Scott Krauss, Richard J Webby, Robert G Webster, Samuel K Martin, Fred Wabwire-Mangen, Mariette F Ducatez

**Affiliations:** 1 College of Veterinary Medicine, Makerere University, P.O. Box 7062, Kampala, Uganda; 2 Makerere University Walter Reed project, P.O. Box 16524, Kampala, Uganda; 3 Ministry of Health, Kampala, Uganda; 4 Uganda Virus Research Institute, Entebbe, Uganda; 5 College of health Sciences Makerere University, Makerere, Uganda; 6 Department of Infectious Diseases, St. Jude Children’s Research Hospital, Memphis, TN, USA; 7 U.S. Army Medical Research Unit-Kenya, U.S. Embassy, Attn: MRU, United Nations Avenue, P.O. Box 606, Village Market 00621, Nairobi, Kenya; 8 INRA UMR 1225 IHAP Interactions hôtes-agents pathogènes, Université de Toulouse, INP, ENVT, Toulouse, France

**Keywords:** Influenza B, Genetic analysis, Uganda

## Abstract

**Background:**

Influenza B viruses can cause morbidity and mortality in humans but due to the lack of an animal reservoir are not associated with pandemics. Because of this, there is relatively limited genetic sequences available for influenza B viruses, especially from developing countries. Complete genome analysis of one influenza B virus and several gene segments of other influenza B viruses isolated from Uganda from May 2009 through December 2010 was therefore undertaken in this study.

**Methods:**

Samples were collected from patients showing influenza like illness and screened for influenza A and B by PCR. Influenza B viruses were isolated on Madin-Darby Canine Kidney cells and selected isolates were subsequently sequenced and analyzed phylogenetically.

**Findings:**

Of the 2,089 samples collected during the period, 292 were positive by PCR for influenza A or B; 12.3% of the PCR positives were influenza B. Thirty influenza B viruses were recovered and of these 25 that grew well consistently on subculture were subjected to further analysis. All the isolates belonged to the B/Victoria-lineage as identified by hemagglutination inhibition assay and genetic analysis except one isolate that grouped with the B-Yamagata-lineage. The Ugandan B/Victoria-lineage isolates grouped in clade 1 which was defined by the N75K, N165K and S172P substitutions in hemagglutinin (HA) protein clustered together with the B/Brisbane/60/2008 vaccine strain. The Yamagata-like Ugandan strain, B/Uganda/MUWRP-053/2009, clustered with clade 3 Yamagata viruses such as B/Bangladesh/3333/2007 which is characterized by S150I and N166Y substitutions in HA.

**Conclusion:**

In general there was limited variation among the Ugandan isolates but they were interestingly closer to viruses from West and North Africa than from neighboring Kenya. Our isolates closely matched the World Health Organization recommended vaccines for the seasons.

## Background

Influenza viruses continue to cause significant morbidity and mortality around the world. Influenza A viruses have attracted a lot of attention because of their potential to cause serious pandemics with the most recent being the 2009 H1N1 virus [1]. Influenza B viruses differ from influenza A viruses by the lack of protein basic 1 – F2 (PB1-F2) but they also have additional proteins that are not found in influenza A viruses such as the glycoprotein B (NB) as well as other differences in the genome [2,3]. In addition, while influenza A viruses have a natural reservoir in waterbirds [4], influenza B viruses do not have an animal natural reservoir although human influenza B viruses have been reported in seals [5], contributing to the virus limited diversity, which explains why they do not cause pandemics [6].

Influenza B viruses are broadly classified into two genetic lineages (B/Victoria/2/87-like and B/Yamagata/16/88-like) that circulate globally with unpredictable temporal and spatial distributions [7]. These viruses have been reported to evolve over time due to selection pressure supplied by pre-existing immunity resulting in changes in antigenic and genetic types [8].

Whole genome data on influenza viruses enables a deeper understanding of the pathogenesis, epidemiology, and drug sensitivities of circulating viruses. While there is an increasing amount of influenza B virus hemagglutinin (HA) gene sequences available, information on the other genes and their role in pathogenesis is still limited. Moreover, more severe cases of influenza B have been reported [9]. With advancement and ease in generating full genome sequences for many organisms [10], it has become critical to examine full genomes of these viruses in order to create a more robust understanding of the evolution and pathogenesis of influenza B viruses to support advances in new technologies for vaccine design and other control strategies. We describe herein the full genome analysis of one influenza B virus and several gene segments of other influenza B viruses isolated in Uganda in 2009 and 2010.

## Results

### Influenza B occurrence and antigenic characterization

The viruses were isolated from 2,089 patients showing influenza-like illness (ILI) as defined (a fever (38°C) plus either a cough or a sore throat within the past 72 hours) between 2009 and 2010. The samples were obtained from outpatients from the Makerere University Walter Reed Project Influenza surveillance hospital sites (Mulago, Jinja, Bugiri, Gulu and Kayunga). During this period, there was co-circulation of influenza A and B viruses. The overall influenza prevalence was 14.0% and 292 influenza viruses were isolated. By PCR, influenza B accounted for 12.3% of the 292 total influenza isolates recovered during that period. In total, 30 influenza B viruses were isolated and confirmed by PCR and immunofluorescence assays. However only 25 isolates that grew consistently well on subculture were subjected to further analysis (Table 1). From July 1^st^ of 2009, the pandemic H1N1 was reported in Uganda and this peaked in October 2009. However the 2009 season was predominated by Flu B while the 2010 season had two peaks; the July peak by H3N2 subtype and the pandemic peak in October 2010 (Additional file 1: Figure S7). The ILI-symptoms were mainly observed in children below 5 years and indeed most of the influenza B isolates were from this age-group. Table 1 summarizes the data on the isolates analyzed in this study. Most of the influenza B viruses tested (96%; 24/25) belonged to Victoria-lineage as shown by their inhibition of antiserum in hemagglutination inhibition (HI) assay with titers of 640–1280 except for B/Uganda/MUWRP-089/2009 (HI titer: 160). The B/Uganda/MUWRP-053/2009 virus belonged to the Yamagata lineage: it cross-reacted with antiserum against the B/Florida/4/2006 strain (Yamagata lineage).

**Table 1:** Ugandan isolates included in this study

**Virus name**	**Collection date**	**Age**	**Sentinel hospital**	**Subtype**^**1**^	**HI titres**	**Genes sequenced**^**2**^
B/Uganda/MUWRP-053/2009	6-May-09	4 years	Mulago	Yam	1280	PA*, HA, NA*,
Uganda/MUWRP-054/2009	2-Jul-09	6 months	Mulago	Vic	1280	PB2, PA, HA, NA, NP, M, NS
B/Uganda/MUWRP-055/2009	7-Jul-09	13 months	Mulago	Vic	1280	PB2, PB1, PA, HA, NA, NS
B/Uganda/MUWRP-056/2009	7-Jul-09	12 years	Mulago	Vic	1280	HA, NA, NP, M, NS
B/Uganda/MUWRP-057/2009	10-Jul-09	4 years	Mulago	Vic	1280	HA, NA, NP, M, NS
B/Uganda/MUWRP-060/2009	27-Jul-09	66 months	Mulago	Vic	1280	HA, NA, NP, NS
B/Uganda/MUWRP-063/2009	28-Jul-09	13 years	Mulago	Vic	1280	PB2, PA, HA, NA, M, NS
B/Uganda/MUWRP-064/2009	29-Jul-09	8 years	Mulago	Vic	1280	PB2, PB1, PA, HA, NA, M, NS
B/Uganda/MUWRP-068/2009	6-Aug-09	18 months	Mulago	Vic	1280	PB2, PB1, PA, HA, NA, NS
B/Uganda/MUWRP-073/2009	13-Aug-09	14 months	Mulago	Vic	1280	PB2, PB1, PA, HA, NA,
B/Uganda/MUWRP-076/2009	20-Aug-09	28 months	Mulago	Vic	1280	HA,
B/Uganda/MUWRP-077/2009	20-Aug-09	35 months	Mulago	Vic	1280	PB2, PB1, HA, NA, M, NS
B/Uganda/MUWRP-078/2009	24-Aug-09	6 months	Mulago	Vic	1280	HA, NA,
B/Uganda/MUWRP-080/2009	25-Aug-09	12 months	Mulago	Vic	1280	PB1, PA, HA, NA, NS
B/Uganda/MUWRP-081/2009	26-Aug-09	35 months	Mulago	Vic	1280	PB2, PB1, PA, HA, NA, NP, M, NS
B/Uganda/MUWRP-083/2009	27-Aug-09	63 months	Mulago	Vic	1280	HA, NA,
B/Uganda/MUWRP-084/2009	27-Aug-09	10 months	Mulago	Vic	1280	HA, NA,
B/Uganda/MUWRP-088/2009	8-Sep-09	8 months	Mulago	Vic	640	HA, NA,
B/Uganda/MUWRP-089/2009	10-Sep-09	52 months	Mulago	Vic	160	HA, NA, NS
B/Uganda/MUWRP-115/2009	21-Oct-09	6 years	Jinja	Vic	1280	HA, NP, M, NS
B/Uganda/MUWRP-122/2009	23-Oct-09	6 years	Bugiri	Vic	1280	HA, NA, NP, M, NS
B/Uganda/MUWRP-136/2009	02-Nov-09	60 months	Jinja	Vic	1280	PB1, PA, HA, NP,
B/Uganda/MUWRP-154/2010	8-Jan-10	21 years	Mulago	Vic	640	HA, NP
B/Uganda/MUWRP-158/2010	24-Mar-10	39 months	Mulago	Vic	640	HA, NA, NP
B/Uganda/MUWRP-237/2010	17-Dec-10	6 months	Mulago	Vic	640	PB1*, PA*, HA, NA,

^1^ Yam: Yamagata lineage, B/Florida/4/2006-like virus; Vic: Victoria lineage, B/Malaysia/2506/2004-like virus for the 2009 isolates and B/Brisbane/60/2008-like virus for the 2010 isolates.

^2^ * only partial sequence available.

### Sequence and phylogenetic analysis

**Figure 1:**
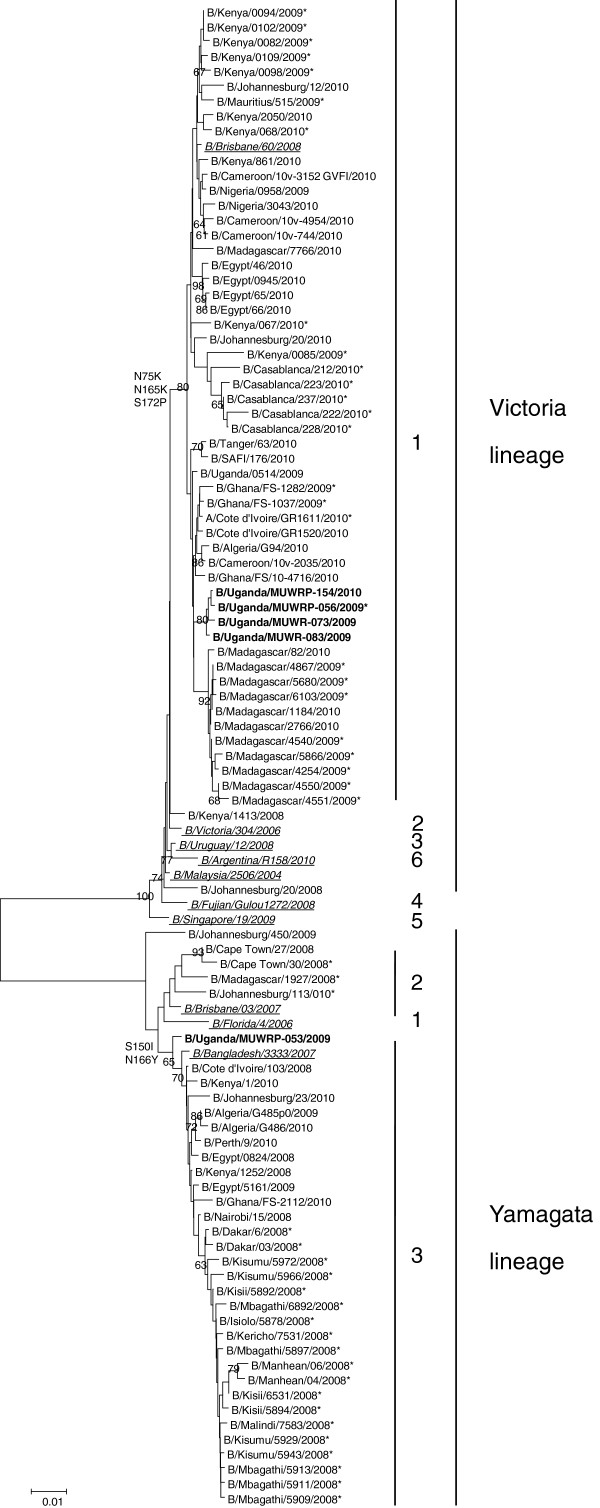
**Phylogenetic tree of the hemagglutinin (HA) gene segment of Ugandan influenza B isolates (in bold font) at the nucleotide level. **The HA sequences of our Ugandan influenza B isolates were compared with relevant virus sequences available in GenBank and GISAID databases: the reference strains for each lineage and group (for the Victorian lineage: B/Brisbane/60/2008, B/Victoria/304/2006, B/Uruguay/12/2008, B/Fujian-Gulou/1272/2008, B/Singapore/19/2009, and B/Argentina/R158/2010 as representatives of group 1, 2, 3, 4, 5, and 6, respectively; for the Yamagata lineage: B/Florida/04/2006, B/Brisbane/03/2007, and B/Bangladesh/3333/2007 as representatives of group 1, 2, and 3, respectively; all represented in italic underlined font), as well as all the African influenza B viruses from 2008 to 2010 available in the databases (a single representative virus was selected for strains with identical amino acid (aa) sequences). B/Uganda/MUWRP-81/2009 was identical to B/Uganda/0514/2009, B/Uganda/MUWRP-54/2009, B/Uganda/MUWRP-55/2009, B/Uganda/MUWRP-57/2009, B/Uganda/MUWRP-60/2009, B/Uganda/MUWRP-63/2009, B/Uganda/MUWRP-64/2009, B/Uganda/MUWRP-68/2009, B/Uganda/MUWRP-76/2009, B/Uganda/MUWRP-77/2009, B/Uganda/MUWRP-78/2009, B/Uganda/MUWRP-80/2009, B/Uganda/MUWRP-84/2009, B/Uganda/MUWRP-115/2009, B/Uganda/MUWRP-122/2009, B/Uganda/MUWRP-136/2009, B/Uganda/MUWRP-158/2010, and B/Uganda/MUWRP-237/2010; B/Uganda/MUWRP-73/2009 to B/Uganda/MUWRP-88/2009 and B/Uganda/MUWRP-89/2009. The number of identical Ugandan isolates is indicated in parenthesis when necessary. Aa substitutions characteristics of the main groups are indicated on the tree nodes. Bootstrap values (1000 replicates) >50 are indicated on the nodes. * indicate partial sequence data.

**Figure 2:**
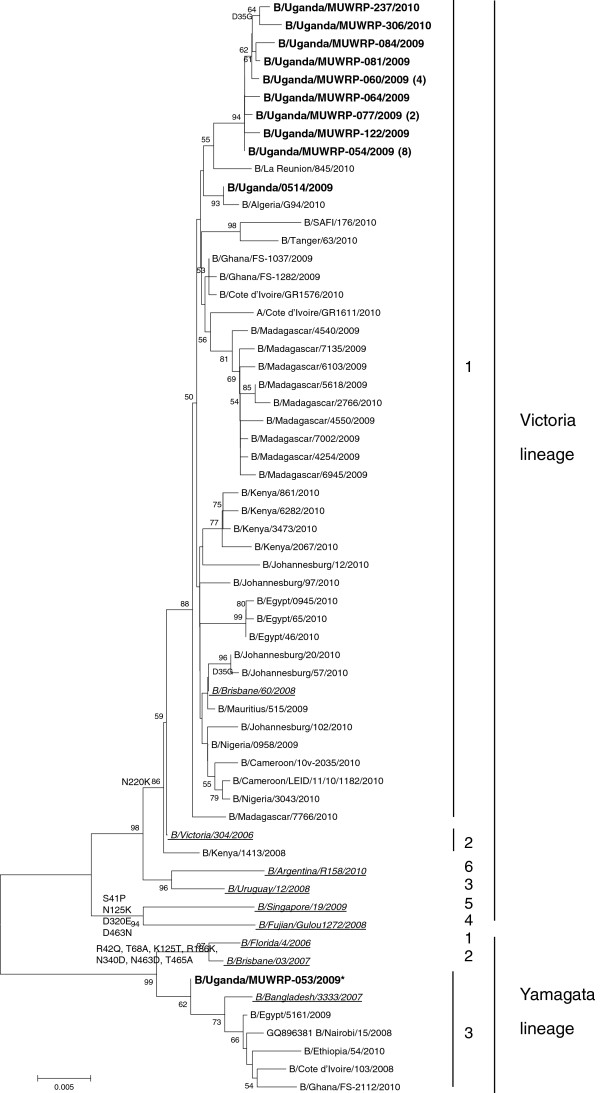
**Phylogenetic tree of the neuraminidase (NA) gene segment of Ugandan influenza B isolates (in bold font) at the nucleotide level. **The NA sequences of our Ugandan influenza B isolates were compared with relevant virus sequences available in GenBank and GISAID databases: the reference strains for each lineage and group (for the Victorian lineage: B/Brisbane/60/2008, B/Victoria/304/2006, B/Uruguay/12/2008, B/Fujian-Gulou/1272/2008, B/Singapore/19/2009, and B/Argentina/R158/2010 as representatives of group 1, 2, 3, 4, 5, and 6, respectively; for the Yamagata lineage: B/Florida/04/2006, B/Brisbane/03/2007, and B/Bangladesh/3333/2007 as representatives of group 1, 2, and 3, respectively; all represented in italic underlined font), as well as all the African influenza B viruses from 2008 to 2010 available on the databases (a single representative virus was selected for strains with identical amino acid (aa) sequences). The NA aa sequence of B/Uganda/MUWRP-054/2009 was identical to the one of B/Uganda/MUWRP-055/2009, B/Uganda/MUWRP-056/2009, B/Uganda/MUWRP-057/2009, B/Uganda/MUWRP-063/2009, B/Uganda/MUWRP-068/2009, B/Uganda/MUWRP-080/2009, and B/Uganda/MUWRP-083/2009; B/Uganda/MUWRP-060/2009 to B/Uganda/MUWRP-073/2009, B/Uganda/MUWRP-088/2009, and B/Uganda/MUWRP-089/2009; B/Uganda/MUWRP-077/2009 to B/Uganda/MUWRP-078/2009; B/Uganda/0519/2009 to B/Uganda/MUWRP-136/2009. The number of identical Ugandan isolates is indicated in parenthesis when necessary. Aa substitutions characteristics of the main groups are indicated on the tree nodes. Bootstrap values (1000 replicates) >50 are indicated on the nodes. * indicate partial sequence data.

All the Ugandan influenza B isolates belonged to the B/Victoria-lineage as shown on Figure 1 except one isolate, confirming the HI results. Although B/Victoria strains have been classified into 6 genetic clades on the basis of their HA sequences, the Uganda influenza B Victoria lineage strains only grouped in genetic clade 1 indicating limited genetic diversity and/or limited introductions of viruses in the country. All B/Victoria-like Ugandan viruses possessed the N75K, N165K and S172P substitutions, characteristics of clade 1 B/Victoria viruses (Figure 1). No amino acid mutation in the HA of B/Uganda/MUWRP-089/2009 could explain its lower inhibition of antiserum raised against B/Malaysia/2506/2004 as compared to its counterparts. The phylogenetic analysis of the neuraminidase (NA) (Figure 2) as well as all of the internal gene segments confirmed this clade 1 grouping (Additional files 2, 3, 4, 5, 6, 7 Figures S1 to S6). Ugandan influenza B isolates from 2009–2010 had residues ^119^F, ^152^R, and ^292^R present on NA for all the sequenced virus strains. The 2009 viruses could be differentiated from the 2010 viruses by a D35G substitution on NA.

A single Ugandan isolate, B/Uganda/MUWRP-053/2009, the earliest isolate reported from the present study; clustered with Yamagata-lineage viruses. Its HA sequence clustered with clade 3 Yamagata viruses such as B/Bangladesh/3333/2007 characterized by S150I and N166Y substitutions (Figure 1). The strain consistently clustered with clade 3 Yamagata-like viruses (Figures 1, 2, and Additional file 4: Figure S3), although genetic sequences could only be obtained for HA, NA, and polymerase acidic (PA) gene segments.

We observed an apparent low variability within Ugandan B/Victoria-like influenza isolates over the study period. The Kimura distance (at the nucleic acid level) ranged between 0 and 1.0% for HA (with the highest Kimura distance between B/Uganda/MUWRP-136/2009 and B/Uganda/MUWRP-237/2010, collected 1 month apart); and between 0.1 and 0.5% for NA (with the highest Kimura distance between B/Uganda/MUWRP-158/2010 and B/Uganda/MUWRP-084/2009 or B/Uganda/MUWRP-122/2009, collected 7 and 5 months apart, respectively). Similarly, pairwise genetic distances at the amino acid level (with a Poisson correction) were very low, ranging between 0 and 0.5% for HA and between 0.2 and 0.7% for NA. Because of the low variability among the isolates, we sequenced and analyzed the full genome of one representative isolate (B/Uganda/MUWRP-081/2009). We also sequenced and analyzed 5 to 7 gene segments for 11 virus strains we worked on (out of a total of 25 isolates). For each isolate, the HA and at least a second gene segment was sequenced. Table 1 summarizes the sequenced genes for each Ugandan isolate. Additional files of sequences used in drawing the phylogenetic trees of all the gene segments have been provided as Fasta files (Additional files 8, 9, 10, 11, 12, 13, 14 and 15: Byarugaba_SFile1-8). The very low genetic diversity observed among the available sequences confirmed the similarities among Ugandan influenza B strains.

## Discussion

The Makerere University Walter Reed Project influenza surveillance was initiated in 2008 following the pandemic threat created by the H5N1 viruses which had exhibited very high mortality rates in humans. Within the period under this study (May 2009 through December 2010), 2,089 samples from patients showing influenza-like illnesses were collected. Although it is still unclear whether the tropics have influenza peak seasons or whether the virus circulates year-round [11], we have previously observed more ILI and corresponding influenza cases around August [12]. This is still true with influenza B and the present study: during this period, the peak of influenza activity in Uganda was observed in July and August (with 16/25 isolated viruses sampled in July or August). The period also coincided with the influenza pandemic due to influenza A(H1N1)pdm09 whose first case in Uganda was reported on July 1^st^ 2009. This period therefore had a co-circulation of A/H3N2, A(H1N1)pdm09 and influenza B viruses.

In 2009, there was co-circulation of Victoria and Yamagata lineages of influenza B viruses observed worldwide [7] that eventually was dominated by the Victoria lineage later in the year as indeed was observed in our study. Co-circulation of the two influenza B viral lineages is a well described phenomenon [13-20]. Interestingly, our unique Yamagata isolate, B/Uganda/MUWRP-053/2009, was our very first isolate (collection date of the original specimen: 6 May 2009, Table 1), which is concordant with a co-circulation of Victoria and Yamagata-like viruses, followed by the dominance of Victoria-like viruses in 2009. The Ugandan influenza B virus sequences were compared with World Health Organization (WHO) vaccine strains recommended for the period of study (WHO weekly epidemiological reports, 2009). Our Yamagata-like isolate of 2009 was antigenically and genetically closely related to the B/Florida/4/2006-like (Yamagata lineage) vaccine strains recommended (WHO weekly epidemiological reports, 2010). Similarly, all our Victoria-like strains showed high antigenic and genetic similarities with the 2009/2010 Northern hemisphere season B/Brisbane/60/2008-like (Victoria lineage) vaccine strain that was recommended for that season. Our study therefore confirms the switch of influenza B lineage in 2009 and suggests a good match between WHO vaccine recommendations and influenza B viruses circulating in Uganda at the time.

Residues for neuraminidase inhibitor susceptibility (^119^E, ^152^R, and ^292^R) have been previously reported for influenza B viruses [21]. All of the Ugandan influenza B isolates characterized in this study had genetic signatures indicating that they remained susceptible to the available neuraminidase inhibitors. It should be noted that Uganda does not have anti-influenza drugs on their essential list of drugs and they are therefore not routinely used by the population. Influenza B viruses with reduced susceptibility to neuraminidase inhibitors have been reported elsewhere [22].

Although our isolates were highly conserved, there was an evolution from the earlier isolates to the most recent strains. The 2009 viruses could be clearly differentiated from the 2010 viruses by a D35G substitution in the neuraminidase protein. We were not able to determine whether this was a within-country evolution or whether it was due to new introductions into the country. However, it was interesting to note that the Victoria-like Ugandan viruses were phylogenetically closer to Northern and Western African viruses than to viruses from their neighboring country, Kenya. All the Ugandan Victoria-like strains grouped in only one genetic subgroup (Clade1) indicating very limited variation among the isolates. The majority of other Victoria-like viruses from elsewhere during the same period also belonged to Clade 1 although a considerable percentage of strains clustered in other clades [7]. The number of isolates and sampling dates may explain the geographical difference. There is no general population vaccination in Uganda (except for specific staff working in influenza laboratories and a few foreign nationals) and this may explain the low genetic diversity observed: influenza viruses evolve with vaccine-induced selection pressure within the country.

## Conclusions

The study confirmed the co-circulation of Victoria and Yamagata lineages of influenza B as was observed elsewhere although the Victoria lineage later dominated in the year. Interestingly, our isolates had limited variation in the genetic makeup, possibly because of no vaccination coverage in the country. Influenza surveillance is important in Uganda as in many African countries where the virus evolution may differ from countries with high vaccine-induced selection pressure.

## Materials and methods

### Sample collection

Samples were collected from five Ugandan hospital-based sentinel surveillance sites in Uganda established by the Makerere University Walter Reed project namely: Mulago National Referral Hospital in Kampala, Kayunga District Hospital, Jinja regional referral Hospital in Jinja District, Bugiri district hospital in Bugiri District, and Gulu regional referral hospital in Gulu District. Nasopharyngeal and throat samples were collected from individuals aged 6 months or older with influenza-like symptoms at the outpatient clinics under routine surveillance for influenza like illness which was ongoing since 2008. The inclusion criteria for enrollment were fever (≥38°C) plus either cough or sore throat within the past 72 hours prior to patient presentation. Any patient too ill to participate or unwilling or unable to provide consent to participate in the surveillance study was excluded. All participants/caretakers were informed about the study and consented to participate in the study by signing (or by a thumbprint if unable to write) a consent form if 18 yrs or older. For children aged less than 8 yrs, their parents/guardians signed the informed consent form while for minors aged 8 to 17 yrs, they together with their parents/guardians signed the consent form. Nasopharangeal and throat swab samples were collected using a dacron swab in a 2-ml cryovial containing virus transport medium. After collection, the samples were immediately stored at −196°C in a liquid nitrogen dry shipper or kept on ice if the samples were to be delivered to the laboratory in less than 8 hours. All samples were transported to Makerere University Walter Reed Influenza Research Laboratories for analysis. The study was approved by the Makerere University School of Public Health Institutional Review Board, the US Army Research and Material Command, and the Uganda National Council for Science and Technology.

### Influenza B screening by RT-PCR

Viral RNA was extracted from all samples by using the QIAamp Viral RNA mini kit (Qiagen) according to the manufacturer’s directions. RT-PCR of the extracts was performed by using a Qiagen One-Step RT-PCR kit according to the manufacturer’s instructions, with the following influenza B matrix gene primers Bfwd: 5^′^ GAGACACAATTGCCTACCTGCTT 3^′^ and Brev: 5^′^ TTCTTTCCCACCGAACCAAC 3^′^ (TAGc, Copenhagen) as described [23]. Briefly, the 25-μl reaction volume contained 5 μl of 5x PCR buffer, 13 μl of RNAse-free H_2_0, 1 μl of 10 mmol/L dNTPs, 1.5 μl of 10 nmol/L reverse primer, 1.5 μl of 10 nmol/L forward primer, 1 μl of enzyme mix (Taq DNA polymerase and reverse transcriptase), and 2 μl of viral RNA extract. Amplification was carried out in an Applied Biosystems Veriti 96-well thermocycler with a single reverse transcription step of 50°C for 30 min, “hot start PCR” (95°C) for 15 sec, forty 30-sec denaturation cycles at 95°C, 30 sec of primer annealing at 55°C, 1 min of extension at 72°C, and further extension for 10 min at 72°C. The samples (including a known positive control) were then separated on a 1% agarose gel with a 50-bp marker. The primers amplified a 250-bp segment of the matrix gene in influenza B–positive samples; this product was visualized and documented in a Biorad Gel Doc XR imager.

### Virus isolation and subtyping

PCR-positive samples (100 μl) were inoculated on 70%-90% confluent Madin-Darby Canine Kidney cell line (MDCK) (NBL2; American Type Culture Collection (ATCC) Rockville, Md.) in flat-sided tubes after pre-treatment with TPCK-trypsin to facilitate virus entry. Tubes were capped loosely, incubated in a tissue culture incubator at 37°C with 5% CO_2_, and observed daily for 10 days for cytopathic effects by light microscopy using an inverted microscope. When cytopathic effects were observed, influenza virus was confirmed by immunofluorescence assay with antibodies against influenza B using Influenza B DFA kits according to the manufacturers (Light Diagnostics) and PCR as above. The isolates were subtyped by HI assay using reference sera obtained from the WHO. All isolates were stored at −80°C until further characterization.

### Sequencing

Sequencing was performed at the Center of Excellence for Influenza Research and Surveillance (CEIRS)/ WHO Collaborating Center for the Ecology of Influenza in Animals at St. Jude Children’s Research Hospital, Memphis, TN. RNA was similarly extracted from 25 isolates isolated between 2009 and 2010 using RNA extraction kits (Qiagen). RT-PCR was carried out for individual gene fragments with primers and PCR conditions as described in Table 2. The different fragments were run on a 1% gel and purified by gel purification kits (Qiagen). Sanger sequencing method was carried out on all the segments using the same primers as used for the PCR.

**Table 2:** Primer sets used for RT-PCR amplification of the eight vRNAs of Ugandan influenza B isolates

**Gene**	**Forward primer**	**Reverse primer**	**Source**
PB1	Bm-PB1b-1: TATTCGTCTCAGGGAGCAGAAGCGGAGCCTTTAAGATG	Bm-PB1b-1200R: TATTCGTCTCGATGCCGTTCCTTCTTCATTGAAGAATGG	^1^
PB1	Bm-PB1b-1220: TATTCGTCTCGGCATCTTTGTCGCCTGGGATGATGATG	Bm-PB1b-2369R: ATATCGTCTCGTATTAGTAGAAACACGAGCCTT	^1^
PB1	BPB1-1103F: AAATACCTTGTCCTGATCTG	BPB1-1685R: TGGCATTTGTAGGTGTATCTAT	^2^
PB2	Bm-PB2b-1: TATTCGTCTCAGGGAGCAGAAGCGGAGCGTTTTCAAGATG	Bm-PB2b-1145R: TATTCGTCTCTCTCATTTTGCTCTTTTTTAATATTCCCC	^1^
PB2	Bm-PB2b-1142: TATTCGTCTCATGAGAATGGAAAAACTACTAATAAATTCAGC	Bm-PB2b-2396R: ATATCGTCTCGTATTAGTAGAAACACGAGCATT	^1^
PB2	BPB2-1045F: GGGAACGGAACAATACAGAAG	BPB2-1320R: CAAAAAATATCGTTGGAGTTG	^2^
PA	Bm-PAb-1: TATTCGTCTCAGGGAGCAGAAGCGGTGCGTTTGA	Bm-PAb-1261R: TATTCGTCTCCCAGGGCCCTTTTACTTGTCAGAGTGC	^1^
PA	Bm-PAb-1283: TATTCGTCTCTCCTGGATCTACCAGAAATAGGGCCAGAC	Bm-PAb-2308R: ATATCGTCTCGTATTAGTAGAAACACGTGCATT	^1^
PA	BPA-510F GGGAGAGTGCTAAGCAGACT	BPA-1430R: TCCCATGCTGGCATTGCTTTCA	^2^
PA	BPA-940F: GAAAAGTACTCAACACTAC	BPA-2020R TCCTTTAATGCTTGAATCAACAG	^2^
HA	BHA-1F: ATGAAGGCAATAATTGTACT	BHA-700R: CATAATGTGTGGTCACTCC	^2^
HA	BHA-673F: CCCCAGAAGTTCACCTCATC	BHA-1742R: CCCTTATAGACAGATGGAGC	^2^
NP	MDV-B 5^′^ BsmBI-NP: TATTCGTCTCAGGGAGCAGAAGCACAGCATTTTCTTGTG	MDV-B 3^′^ BsmBI-NP: ATATCGTCTCGTATTAGTAGAAACAACAGCATTTTTTAC	^1^
NA	Bm-NAb-1: TATTCGTCTCAGGGAGCAGAAGCAGAGCA	Bm-NAb-1557R: ATATCGTCTCGTATTAGTAGTAACAAGAGCATTTT	^1^
NA	BNA-716F: GGGGAAATTGTTATCTTA	BNA-1410R: CCATTCCTCCATTACAGAGCC	^2^
M	MDV-B 5^′^ BsmBI-M: TATTCGTCTCAGGGAGCAGAAGCACGCACTTTCTTAAAATG	MDV-B 3^′^ BsmBI-M: ATATCGTCTCGTATTAGTAGAAACAACGCACTTTTTCCAG	^1^
NS	MDV-B 5^′^ BsmBI-NS: TATTCGTCTCAGGGAGCAGAAGCAGAGGATTTGTTTAGTC	MDV-B 3^′^ BsmBI-NS: ATATCGTCTCGTATTAGTAGTAACAAGAGGATTTTTAT	^1^

^1^[24].

^2^ this study.

### Sequence analysis

The sequences obtained were analyzed using Bioedit Software version 5.0.9 and aligned with Clustal W [25]. Phylogenetic analysis was performed using the Neighbor-Joining algorithm and pairwise deletion using the MEGA version 5.05 program [26] and trees were un-rooted. The number of bootstrap replications was set to 1,000, and bootstrap values above 50 were labeled on major tree branches for reference. The Ugandan virus strains were clustered on the basis of nucleotides, and only dominant clusters were used to infer phylogenetic relationships. The analysis included sequences from representative isolates from different regions of the world available in GenBank and the sequences of both Yamagata and Victoria lineage vaccine strains recommended by WHO for both the southern and northern hemispheres for the influenza seasons from 2008 to 2010. The latter viruses were also used as references to compare the amino acid substitutions seen in the Ugandan sequences. The sequences viruses used in the phylogenetic tress are presented in Additional files 1, 2, 3, 4, 5, 6, 7, and 8 representing sequences used for Figures 1 and 2 and Additional files 2, 3, 4, 5, 6, 7 Figures S1–S6 respectively.

### Accession numbers

The complete gene sequences of the isolates analyzed in this study were deposited in Genbank with accession numbers JX427060- JX427104. The isolates with exactly similar sequences were not included in the Genbank instead they were represented by one sequence and they have been indicated in the phylogenetic tree legends.

## Abbreviations

MDCK: Madin-Darby Canine Kidney; RT: Reverse Transcription; PCR: Polymerase Chain Reaction; WHO: World Health Organization; GISAID: Global Initiative in Sharing Avian Influenza Data; DOD-GEIS: Department of Defense’s Global Emerging Infections Surveillance and Response System.

## Competing interests

The authors declare that they have no competing interest.

## Authors’ contributions

DKB, SKM, FW, EAM, MM, HK, JB, LL conceived and designed the study; DKB, BE,DM, EAM, MM, HK, JB, SKM, FW, LL were involved in the sample collection and virus isolation while DKB, MFD, SB, RJW, SK, RGW were involved in the genetic analysis. DKB, MFD, BE, EAM, MM, HK, JB, SB, SK,SKM, RJW, RGW, FWM and LL contributed to data analysis and writing the paper. All authors read and approved the final manuscript.

## Supplementary Material

Additional file 1 **Figure S7. **Seasonality of influenza in Uganda during the May 2009 to Dec 2010 season. Click here for file

Additional file 2 **Figure S1. **Phylogenetic tree of the protein basic 2 (PB2) gene segment of Ugandan influenza B isolates (in bold font) at the nucleotide level. The PB2 sequences of our Ugandan influenza B isolates were compared with relevant virus sequences available on GenBank and GISAID databases: the available reference strains (for the Victorian lineage: B/Brisbane/60/2008 and B/Fujian-Gulou/1272/2008, as representatives of group 1 and 4, respectively; for the Yamagata lineage: B/Florida/04/2006 and B/Bangladesh/3333/2007 as representatives of group 1 and 3, respectively; all represented in italic underlined font). No 2008–2010 African influenza B viruses PB2 gene sequence was available on the databases. Bootstrap values (1000 replicates) >50 are indicated on the nodes. Click here for file

Additional file 3 **Figure S2. **Phylogenetic tree of the protein basic 1 (PB1) gene segment of Ugandan influenza B isolates (in bold font) at the nucleotide level. The PB1 sequences of our Ugandan influenza B isolates were compared with relevant virus sequences available on GenBank and GISAID databases: the available reference strains (for the Victorian lineage: B/Brisbane/60/2008 and B/Fujian-Gulou/1272/2008, as representatives of group 1 and 4, respectively; for the Yamagata lineage: B/Florida/04/2006 and B/Bangladesh/3333/2007 as representatives of group 1 and 3, respectively; all represented in italic underlined font). No 2008–2010 African influenza B viruses PB1 gene sequence was available on the databases. The aa sequences of B/Uganda/MUWRP-055/2009, B/Uganda/MUWRP-068/2009, B/Uganda/MUWRP-073/2009, B/Uganda/MUWRP-080/2009, and B/Uganda/MUWRP-081/2009 were identical and only B/Uganda/MUWRP-081/2009 is shown on the tree. Bootstrap values (1000 replicates) >50 are indicated on the nodes. * indicate partial sequence data. Click here for file

Additional file 4 **Figure S3. **Phylogenetic tree of the protein acidic (PA) gene segment of Ugandan influenza B isolates (in bold font) at the nucleotide level. The PA sequences of our Ugandan influenza B isolates were compared with relevant virus sequences available on GenBank and GISAID databases: the available reference strains (for the Victorian lineage: B/Brisbane/60/2008 and B/Fujian-Gulou/1272/2008, as representatives of group 1 and 4, respectively; for the Yamagata lineage: B/Florida/04/2006 and B/Bangladesh/3333/2007 as representatives of group 1 and 3, respectively; all represented in italic underlined font). No 2008–2010 African influenza B viruses PA gene sequence was available on the databases. The aa sequences of B/Uganda/MUWRP-055/2009 and B/Uganda/MUWRP-080/2009 were identical and only B/Uganda/MUWRP-055/2009 is shown on the tree. Bootstrap values (1000 replicates) >50 are indicated on the nodes. * indicate partial sequence data. Click here for file

Additional file 5 **Figure S4. **Phylogenetic tree of the nucleoprotein (NP) gene segment of Ugandan influenza B isolates (in bold font) at the nucleotide level. The NP sequences of our Ugandan influenza B isolates were compared with relevant virus sequences available on GenBank and GISAID databases: the available reference strains (for the Victorian lineage: B/Brisbane/60/2008 and B/Fujian-Gulou/1272/2008, as representatives of group 1 and 4, respectively; for the Yamagata lineage: B/Florida/04/2006 and B/Bangladesh/3333/2007 as representatives of group 1 and 3, respectively; all represented in italic underlined font). No 2008–2010 African influenza B viruses NP gene sequence was available on the databases. The aa sequences of B/Uganda/MUWRP-056/2009, B/Uganda/MUWRP-057/2009, B/Uganda/MUWRP-060/2009, B/Uganda/MUWRP-122/2009, and B/Uganda/MUWRP-032/2010 were identical and only B/Uganda/MUWRP-056/2009 is shown on the tree. Bootstrap values (1000 replicates) >50 are indicated on the nodes. Click here for file

Additional file 6 **Figure S5. **Phylogenetic tree of the matrix protein (M) gene segment of Ugandan influenza B isolates (in bold font) at the nucleotide level. The M sequences of our Ugandan influenza B isolates were compared with relevant virus sequences available on GenBank and GISAID databases: the available reference strains (for the Victorian lineage: B/Brisbane/60/2008 and B/Fujian-Gulou/1272/2008, as representatives of group 1 and 4, respectively; for the Yamagata lineage: B/Florida/04/2006 and B/Bangladesh/3333/2007 as representatives of group 1 and 3, respectively; all represented in italic underlined font), as well as all the African influenza B viruses from 2008 to 2010 available on the databases: B/Egypt/0945/2010, B/Kenya/2050/2010, and B/Kenya/2067/2010. The aa sequences of B/Uganda/MUWRP-054/2009, B/Uganda/MUWRP-056/2009, B/Uganda/MUWRP-057/2009, B/Uganda/MUWRP-063/2009, B/Uganda/MUWRP-064/2009, B/Uganda/MUWRP-077/2009, and B/Uganda/MUWRP-122/2009 were identical and only B/Uganda/MUWRP-054/2009 is shown on the tree. The number of identical Ugandan isolates is indicated in parenthesis when necessary. Bootstrap values (1000 replicates) >50 are indicated on the nodes. Click here for file

Additional file 7 **Figure S6. **Phylogenetic tree of the non-structural protein (NS) gene segment of Ugandan influenza B isolates (in bold font) at the nucleotide level. The NS sequences of our Ugandan influenza B isolates were compared with relevant virus sequences available on GenBank and GISAID databases: the available reference strains (for the Victorian lineage: B/Brisbane/60/2008, B/Fujian-Gulou/1272/2008, B/Singapore/19/2009, and B/Argentina/158/2010, as representatives of group 1, 4, 5, and 6, respectively; for the Yamagata lineage: B/Florida/04/2006 and B/Bangladesh/3333/2007 as representatives of group 1 and 3, respectively; all represented in italic underlined font), as well as all the African influenza B viruses from 2008 to 2010 available on the databases. A single representative virus was selected for strains with identical amino acid (aa) sequences: the NS aa sequence of B/Uganda/MUWRP-081/2009 was identical to the one of B/Uganda/MUWRP-054/2009, B/Uganda/MUWRP-055/2009, B/Uganda/MUWRP-057/2009, B/Uganda/MUWRP-060/2009, B/Uganda/MUWRP-063/2009, B/Uganda/MUWRP-068/2009, B/Uganda/MUWRP-077/2009, B/Uganda/MUWRP-080/2009, B/Uganda/MUWRP-089/2009, B/Uganda/MUWRP-115/2009, and B/Uganda/MUWRP-122/2009. The number of identical Ugandan isolates is indicated in parenthesis when necessary. Bootstrap values (1000 replicates) >50 are indicated on the nodes. Click here for file

Additional file 8 **Byarugaba_SFile 1. **Sequences of representative isolates used for drawing the phylogenetic tree of the haemagglutinin protein (HA) gene segment in Figure 1. Click here for file

Additional file 9 **Byarugaba_SFile 2. **Sequences of representative isolates used for drawing the phylogenetic tree of the neuraminidase protein (NA) gene segment in Figure 2. Click here for file

Additional file 10 **Byarugaba_SFile 3. **Sequences of representative isolates used for drawing the phylogenetic tree of the protein basic 2 (PB2) gene segment of PPTX additional file 2 Figure S1. Click here for file

Additional file 11 **Byarugaba_SFile 4. **Sequences of representative isolates used for drawing the phylogenetic tree of the protein basic 1 (PB1) gene segment of PPTX additional file 3 Figure S2. Click here for file

Additional file 12 **Byarugaba_SFile 5. **Sequences of representative isolates used for drawing the phylogenetic tree of the protein acidic (PA) gene segment of PPTX additional file 4 Figure S3. Click here for file

Additional file 13 **Byarugaba_SFile 6. **Sequences of representative isolates used for drawing the phylogenetic tree of the nucleoprotein (NP) gene segment of PPTX additional file 5 Figure S4. Click here for file

Additional file 14 **Byarugaba_SFile 7. **Sequences of representative isolates used for drawing the phylogenetic tree of the matrix protein (M) gene segment of PPTX additional file 6 Figure S5. Click here for file

Additional file 15 **Byarugaba_SFile 8. **Sequences of representative isolates used for drawing the phylogenetic tree of the non-structural protein (NS) gene segment of PPTX additional file 7 Figure S6. Click here for file
