# Supplementary figures and images for: Genetic analysis of influenza B viruses isolated in Uganda during the 2009–2010 seasons
Source: Virol J. 2013 Jan 5;10:11. doi: 10.1186/1743-422X-10-11 (PMC3547786; doi:10.1186/1743-422X-10-11)

## Slide 1
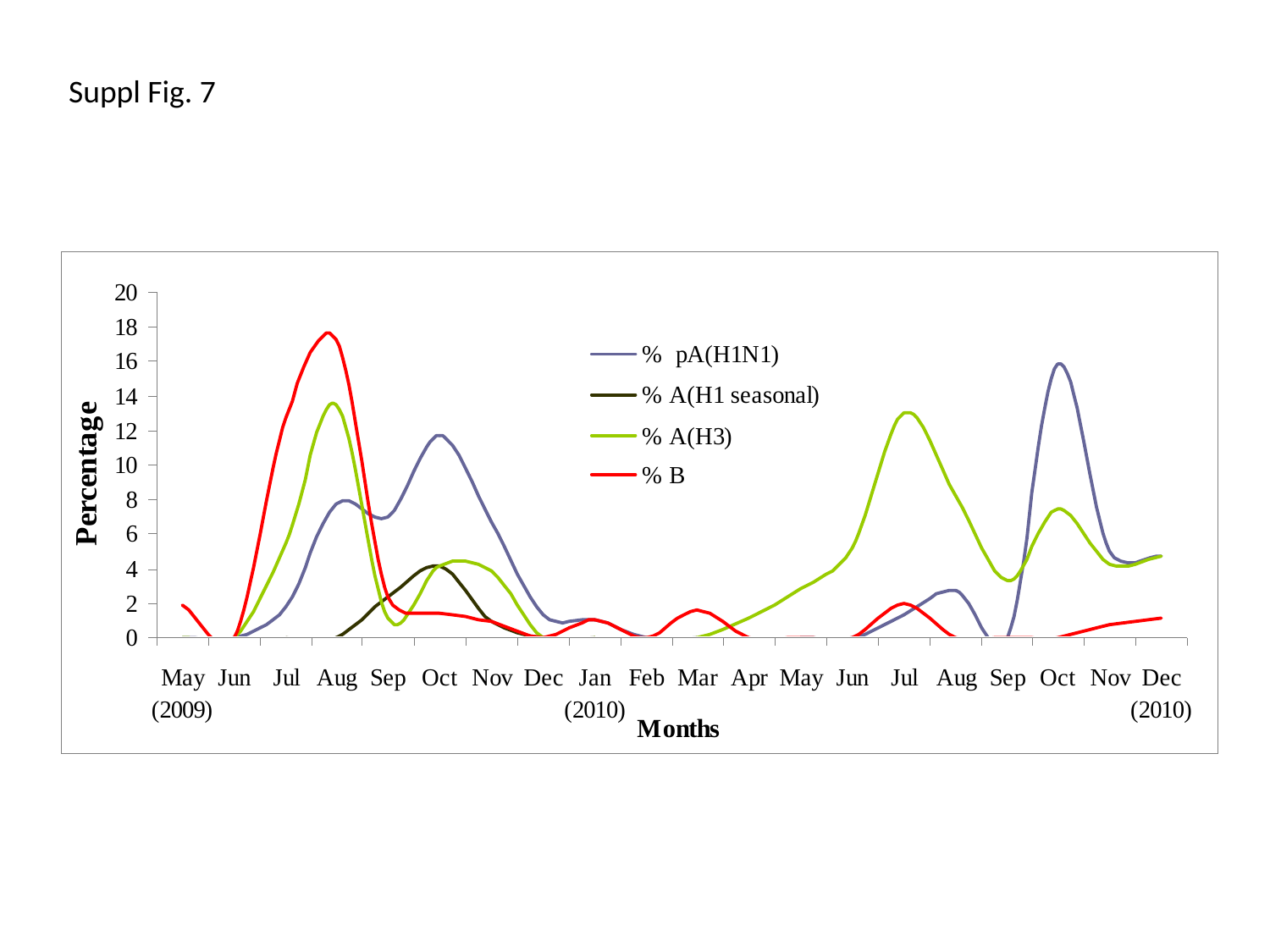

Suppl Fig. 7

Supplement: Additional file 1 — Figure S7. Seasonality of influenza in Uganda during the May 2009 to Dec 2010 season. [file 1743-422X-10-11-S1.pptx]
