# Supplementary material for: Genetic analysis of influenza B viruses isolated in Uganda during the 2009–2010 seasons
Source: Virol J. 2013 Jan 5;10:11. doi: 10.1186/1743-422X-10-11 (PMC3547786; doi:10.1186/1743-422X-10-11)
Supplement: Additional file 4 — Figure S3. Phylogenetic tree of the protein acidic (PA) gene segment of Ugandan influenza B isolates (in bold font) at the nucleotide level. The PA sequences of our Ugandan influenza B isolates were compared with relevant virus sequences available on GenBank and GISAID databases: the available reference strains (for the Victorian lineage: B/Brisbane/60/2008 and B/Fujian-Gulou/1272/2008, as representatives of group 1 and 4, respectively; for the Yamagata lineage: B/Florida/04/2006 and B/Bangladesh/3333/2007 as representatives of group 1 and 3, respectively; all represented in italic underlined font). No 2008–2010 African influenza B viruses PA gene sequence was available on the databases. The aa sequences of B/Uganda/MUWRP-055/2009 and B/Uganda/MUWRP-080/2009 were identical and only B/Uganda/MUWRP-055/2009 is shown on the tree. Bootstrap values (1000 replicates) >50 are indicated on the nodes. * indicate partial sequence data. [file 1743-422X-10-11-S4.pptx]

## Slide 1
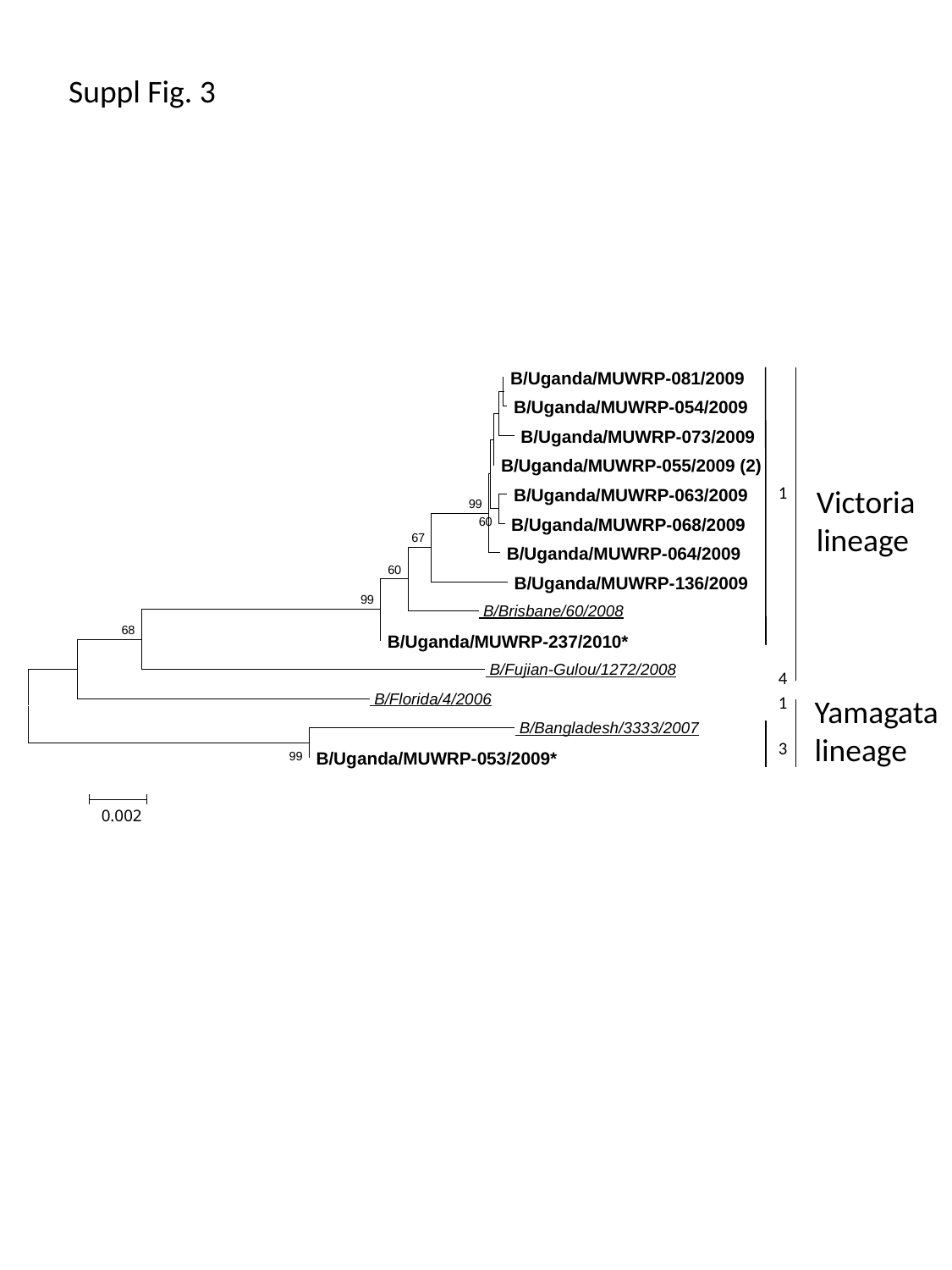

Suppl Fig. 3
 B/Uganda/MUWRP-081/2009
 B/Uganda/MUWRP-054/2009
 B/Uganda/MUWRP-073/2009
 B/Uganda/MUWRP-055/2009 (2)
 B/Uganda/MUWRP-063/2009
99
 B/Uganda/MUWRP-068/2009
60
67
 B/Uganda/MUWRP-064/2009
60
 B/Uganda/MUWRP-136/2009
99
 B/Brisbane/60/2008
68
 B/Uganda/MUWRP-237/2010*
 B/Fujian-Gulou/1272/2008
 B/Florida/4/2006
 B/Bangladesh/3333/2007
 B/Uganda/MUWRP-053/2009*
99
0.002
1
Victoria lineage
4
1
Yamagata lineage
3
