# Supplementary material for: Genetic analysis of influenza B viruses isolated in Uganda during the 2009–2010 seasons
Source: Virol J. 2013 Jan 5;10:11. doi: 10.1186/1743-422X-10-11 (PMC3547786; doi:10.1186/1743-422X-10-11)
Supplement: Additional file 5 — Figure S4. Phylogenetic tree of the nucleoprotein (NP) gene segment of Ugandan influenza B isolates (in bold font) at the nucleotide level. The NP sequences of our Ugandan influenza B isolates were compared with relevant virus sequences available on GenBank and GISAID databases: the available reference strains (for the Victorian lineage: B/Brisbane/60/2008 and B/Fujian-Gulou/1272/2008, as representatives of group 1 and 4, respectively; for the Yamagata lineage: B/Florida/04/2006 and B/Bangladesh/3333/2007 as representatives of group 1 and 3, respectively; all represented in italic underlined font). No 2008–2010 African influenza B viruses NP gene sequence was available on the databases. The aa sequences of B/Uganda/MUWRP-056/2009, B/Uganda/MUWRP-057/2009, B/Uganda/MUWRP-060/2009, B/Uganda/MUWRP-122/2009, and B/Uganda/MUWRP-032/2010 were identical and only B/Uganda/MUWRP-056/2009 is shown on the tree. Bootstrap values (1000 replicates) >50 are indicated on the nodes. [file 1743-422X-10-11-S5.pptx]

## Slide 1
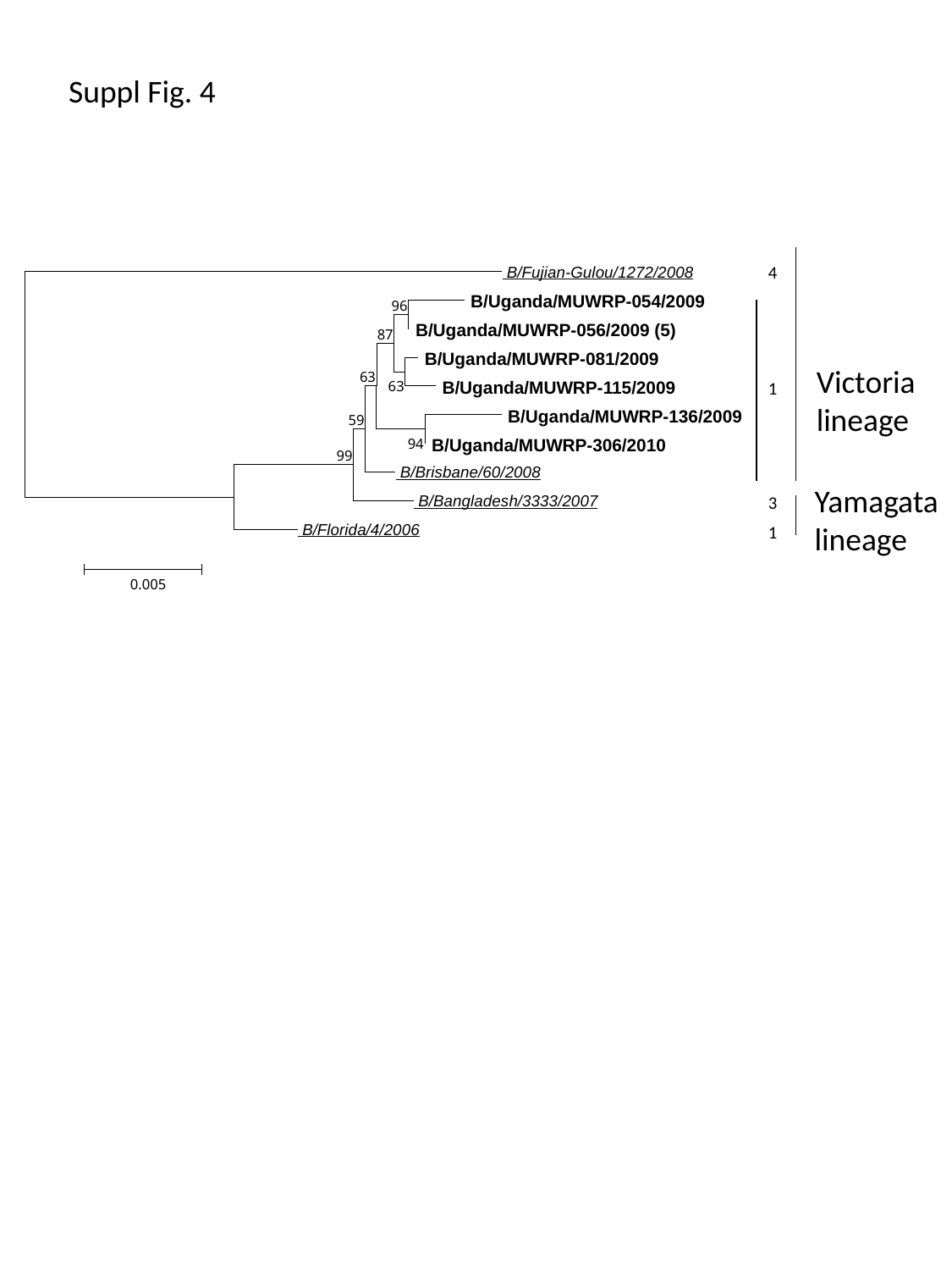

Suppl Fig. 4
 B/Fujian-Gulou/1272/2008
 B/Uganda/MUWRP-054/2009
96
 B/Uganda/MUWRP-056/2009 (5)
87
 B/Uganda/MUWRP-081/2009
63
 B/Uganda/MUWRP-115/2009
63
 B/Uganda/MUWRP-136/2009
59
 B/Uganda/MUWRP-306/2010
94
99
 B/Brisbane/60/2008
 B/Bangladesh/3333/2007
 B/Florida/4/2006
0.005
4
Victoria lineage
1
Yamagata lineage
3
1
