# Supplementary material for: Genetic analysis of influenza B viruses isolated in Uganda during the 2009–2010 seasons
Source: Virol J. 2013 Jan 5;10:11. doi: 10.1186/1743-422X-10-11 (PMC3547786; doi:10.1186/1743-422X-10-11)
Supplement: Additional file 6 — Figure S5. Phylogenetic tree of the matrix protein (M) gene segment of Ugandan influenza B isolates (in bold font) at the nucleotide level. The M sequences of our Ugandan influenza B isolates were compared with relevant virus sequences available on GenBank and GISAID databases: the available reference strains (for the Victorian lineage: B/Brisbane/60/2008 and B/Fujian-Gulou/1272/2008, as representatives of group 1 and 4, respectively; for the Yamagata lineage: B/Florida/04/2006 and B/Bangladesh/3333/2007 as representatives of group 1 and 3, respectively; all represented in italic underlined font), as well as all the African influenza B viruses from 2008 to 2010 available on the databases: B/Egypt/0945/2010, B/Kenya/2050/2010, and B/Kenya/2067/2010. The aa sequences of B/Uganda/MUWRP-054/2009, B/Uganda/MUWRP-056/2009, B/Uganda/MUWRP-057/2009, B/Uganda/MUWRP-063/2009, B/Uganda/MUWRP-064/2009, B/Uganda/MUWRP-077/2009, and B/Uganda/MUWRP-122/2009 were identical and only B/Uganda/MUWRP-054/2009 is shown on the tree. The number of identical Ugandan isolates is indicated in parenthesis when necessary. Bootstrap values (1000 replicates) >50 are indicated on the nodes. [file 1743-422X-10-11-S6.pptx]

## Slide 1
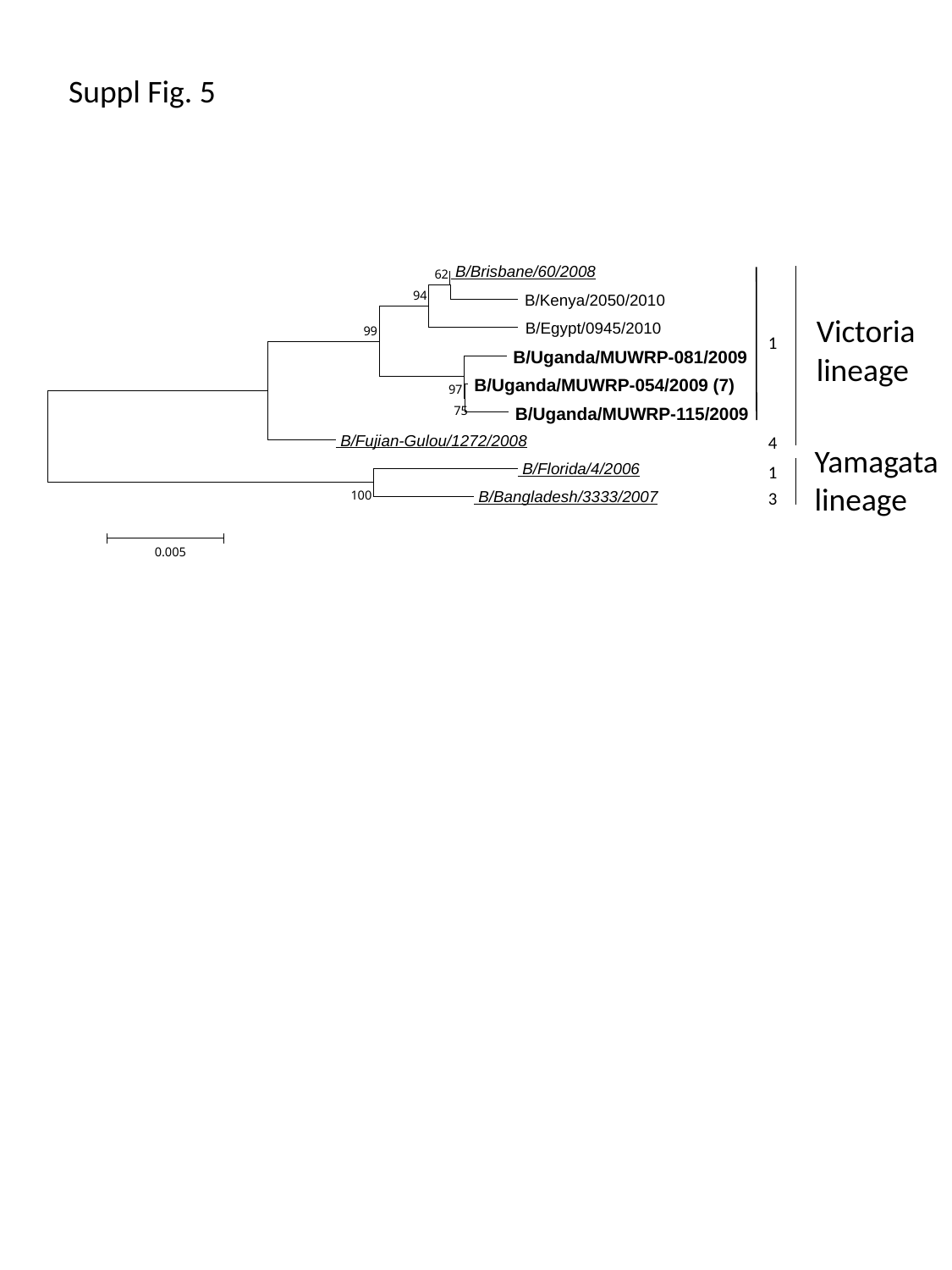

Suppl Fig. 5
 B/Brisbane/60/2008
62
94
 B/Kenya/2050/2010
 B/Egypt/0945/2010
99
 B/Uganda/MUWRP-081/2009
 B/Uganda/MUWRP-054/2009 (7)
97
 B/Uganda/MUWRP-115/2009
75
 B/Fujian-Gulou/1272/2008
 B/Florida/4/2006
 B/Bangladesh/3333/2007
100
0.005
Victoria lineage
1
4
Yamagata lineage
1
3
