# Supplementary material for: Genetic analysis of influenza B viruses isolated in Uganda during the 2009–2010 seasons
Source: Virol J. 2013 Jan 5;10:11. doi: 10.1186/1743-422X-10-11 (PMC3547786; doi:10.1186/1743-422X-10-11)
Supplement: Additional file 7 — Figure S6. Phylogenetic tree of the non-structural protein (NS) gene segment of Ugandan influenza B isolates (in bold font) at the nucleotide level. The NS sequences of our Ugandan influenza B isolates were compared with relevant virus sequences available on GenBank and GISAID databases: the available reference strains (for the Victorian lineage: B/Brisbane/60/2008, B/Fujian-Gulou/1272/2008, B/Singapore/19/2009, and B/Argentina/158/2010, as representatives of group 1, 4, 5, and 6, respectively; for the Yamagata lineage: B/Florida/04/2006 and B/Bangladesh/3333/2007 as representatives of group 1 and 3, respectively; all represented in italic underlined font), as well as all the African influenza B viruses from 2008 to 2010 available on the databases. A single representative virus was selected for strains with identical amino acid (aa) sequences: the NS aa sequence of B/Uganda/MUWRP-081/2009 was identical to the one of B/Uganda/MUWRP-054/2009, B/Uganda/MUWRP-055/2009, B/Uganda/MUWRP-057/2009, B/Uganda/MUWRP-060/2009, B/Uganda/MUWRP-063/2009, B/Uganda/MUWRP-068/2009, B/Uganda/MUWRP-077/2009, B/Uganda/MUWRP-080/2009, B/Uganda/MUWRP-089/2009, B/Uganda/MUWRP-115/2009, and B/Uganda/MUWRP-122/2009. The number of identical Ugandan isolates is indicated in parenthesis when necessary. Bootstrap values (1000 replicates) >50 are indicated on the nodes. [file 1743-422X-10-11-S7.pptx]

## Slide 1
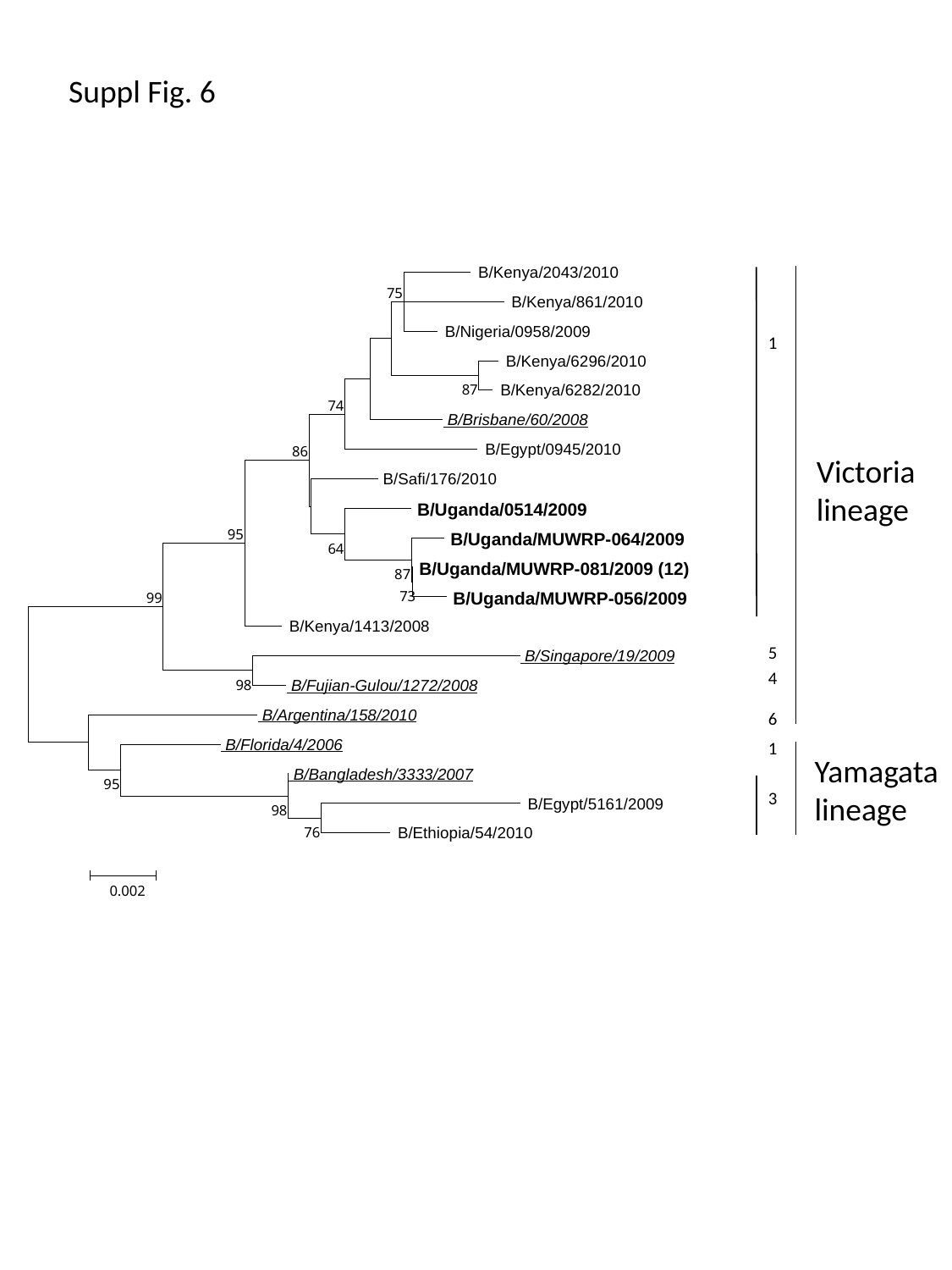

Suppl Fig. 6
 B/Kenya/2043/2010
75
 B/Kenya/861/2010
 B/Nigeria/0958/2009
 B/Kenya/6296/2010
 B/Kenya/6282/2010
87
74
 B/Brisbane/60/2008
 B/Egypt/0945/2010
86
 B/Safi/176/2010
 B/Uganda/0514/2009
95
 B/Uganda/MUWRP-064/2009
64
 B/Uganda/MUWRP-081/2009 (12)
87
 B/Uganda/MUWRP-056/2009
73
99
 B/Kenya/1413/2008
 B/Singapore/19/2009
 B/Fujian-Gulou/1272/2008
98
 B/Argentina/158/2010
 B/Florida/4/2006
 B/Bangladesh/3333/2007
95
 B/Egypt/5161/2009
98
 B/Ethiopia/54/2010
76
0.002
1
Victoria lineage
5
4
6
1
Yamagata lineage
3
